# Possible uses of Hunter–Schreger bands of dental enamel for automated personal identification

**DOI:** 10.1186/s40001-024-01698-7

**Published:** 2024-02-04

**Authors:** Mario Dioguardi, Lorenzo Sanesi, Diego Sovereto, Andrea Ballini, Vito Crincoli, Mario Alovisi, Riccardo Aiuto, Giorgia Apollonia Caloro, Lorenzo Lo Muzio

**Affiliations:** 1 Department of Clinical and Experimental Medicine, University of Foggia, Via Rovelli 50, 71122 Foggia, Italy; 2 Department of Basic Medical Sciences, Neurosciences and Sensory Organs, Division of Complex Operating Unit of Dentistry, “Aldo Moro” University of Bari, Piazza G. Cesare 11, 70124 Bari, Italy; 3 Department of Surgical Sciences, Dental School, University of Turin, 10127 Turin, Italy; 4 Department of Biomedical, Surgical, and Dental Science, University of Milan, 20122 Milan, Italy; 5 Unità Operativa Nefrologia e Dialisi, Presidio Ospedaliero Scorrano, ASL (Azienda Sanitaria Locale) Lecce, Via Giuseppina Delli Ponti, 73020 Scorrano, Italy

**Keywords:** Hunter–Schreger bands, Personal identification, Enamel, Dental

## Abstract

**Background:**

Hunter–Schreger bands (HSB) are optical phenomena observed on tooth surfaces under polarized light, resulting from the intersection of enamel prisms. Anthropological studies demonstrate the prevalence of HSB in large mammals, contributing to enamel resistance. Historically, John Hunter and Schreger depicted HSB in dental literature. In dentistry, HSB play a role in non-carious cervical lesions (NCCL) and internal dental perikymata, suggesting their potential for personal identification. Personal identification, crucial in both daily and professional life, involves biometric characteristics, such as fingerprints or facial recognition. The need for non-invasive, rapid, and user-friendly methods has prompted the exploration of using HSB dental images for personal identification. The review aimed to consolidate studies employing HSB for personal identification.

**Methods:**

The scoping review was carried out strictly following the PRISMA–ScR checklist; the search was carried out on tree databases (PubMed, Scopus, Science Direct,) and a register (Cochrane library).

**Results:**

The research produced a number of bibliographic sources totaling 410. With the removal of duplicates, 334 were obtained; potentially eligible articles amounted to 14, of which only 4 fully complied with the criteria of eligibility.

**Conclusions:**

From the data in the literature, we can assert that HSB could be used in personal identification, as they are characteristics that are difficult to change and easily detectable.

## Introduction

The Hunter–Schreger bands (HSB) result from an optical phenomenon observed when a dental surface, composed of enamel, is sectioned and examined under polarized light. Reflected light rays reveal an alternating pattern of darker bands juxtaposed with lighter bands. The visual perception of the bands as dark (unipolar reflecting) or light (birefringent) depends on the angle of incidence of polarized light. These bands represent the structural manifestation arising from the synchronous intersection of groups of enamel prisms in the horizontal plane, originating from irregularities in the orientation of prism axes.

Anthropological and evolutionary studies indicate that Hunter–Schreger bands (HSB) are predominantly present in large-sized mammals, serving to enhance enamel resistance and prevent the formation of cracks. Among the earliest depictions of HSB, we find them in John Hunter’s text, ‘The Natural History of the Human Teeth,’ published in 1771, with observations later confirmed by Schreger in 1800 [1].

In conservative dentistry, Hunter–Schreger bands (HSB) assume significance due to their role in the genesis of non-carious cervical lesions (NCCL) [2]. Furthermore, it is posited that they bear responsibility for internal dental perikymata (where external are primarily dental perikymata influenced by Retzius’ striae) [3], potentially observable on the external surface through photographic imaging [4]. Both internal and external dental perikymata a have recently been proposed as tools for personal identification [5].

Personal identification is of paramount importance in both everyday life and professional settings. Consider access tools to personal computers and smartphones, which heavily rely on access codes and biometric features, such as fingerprints or facial recognition. Individual biometric measurements must, therefore, be unequivocal and distinguishable among different individuals. Parameters such as skin color, hair color, iris characteristics, height, and weight are easily alterable and can vary over time. Moreover, the methodology for information acquisition and transfer must be non-invasive, rapid, and preferably user-friendly [6].

The visualization of Hunter–Schreger bands (HSB) could address this need, as indicated by Ramenzoni et al., highlighting how the analysis of the optical imprint of the tooth is non-invasive, accurate, and can be easily conducted in automated systems [7].

Currently, in the scientific literature, there are studies that have explored the potential of automated systems leveraging the unique patterns of HSB for human identification. Automated dental recognition systems employ advanced imaging and pattern recognition technologies to analyze the distinctive arrangements of HSB, offering a non-invasive and potentially accurate method for identifying individuals based on their dental features. This emerging field shows promise for applications in forensic science and biometrics, providing a novel approach to human identification through the analysis of dental microstructures, such as HSB [8].

At present, there are no specific automated systems for human identification based solely on HSB. However, in the fields of forensic science and biometrics, where dental analysis can be utilized for identification purposes, there are systems that employ high-resolution dental images. These systems often utilize advanced feature recognition algorithms and may, in the future, integrate HSB as one of the analyzed elements. Currently, most automated human identification systems rely on broader dental features, such as overall shape, size, and arrangement of teeth, rather than specifically on HSB [7].

The aim of this review is to summarize all studies that have employed HSB for personal identification and individual recognition, to assess whether, given the current state of knowledge, there is a possibility of utilizing HSB.

## Materials and methods

### Protocol and registration

The scope review was written and performed following the PRISMA–ScR (PRISMA Extension for Scoping Reviews) checklist as reported by Tricco et al. [9]; Although the review protocol was prepared before the database search was executed, it was deemed appropriate not to register it.

### Eligibility criteria

All studies examining Hunter–Schreger bands in relation to personal identification were considered potentially eligible. There were no restrictions based on the publication year, and language, provided that an English abstract was available. Literature reviews were excluded and solely utilized as sources for bibliographic research.

### Information sources

The search was conducted across three databases (PubMed, Scopus, and Science Direct) and a registry (Cochrane Library). In addition, grey literature searches were performed on Google Scholar and Opengray (DANS EASY Archive). Potentially eligible articles were also sought among the references from literature reviews on Hunter–Schreger bands.

The search was carried out from October 15, 2023, to November 6, 2023, with the latest update of identified records on November 8, 2023.

### Search

The authors responsible for researching the studies used the following key words in the databases: Hunter–Schreger bands The key words used on PubMed are shown below; Search: Hunter–Schreger bands Sort by: Most Recent, (“hunter”[All Fields] OR “hunter s”[All Fields] OR “hunters”[All Fields]) AND “schreger”[All Fields] AND (“band s”[All Fields] OR “bands”[All Fields]), Translations; Hunter: “hunter”[All Fields] OR “hunter’s”[All Fields] OR “hunters”[All Fields], bands: “band’s”[All Fields] OR “bands”[All Fields.

### Selection of sources of evidence

The search for eligible articles and reports was a collaborative effort between two reviewers, M.D. and G.A.C., with a third reviewer, A.B., assigned to resolve conflicts and make inclusion decisions. The primary reviewers, after reaching a consensus on eligibility criteria, keywords, and selected databases, independently conducted the search for articles and reports. They meticulously recorded the number of articles retrieved for each keyword and from each designated database. Duplicate studies identified across different databases were systematically eliminated using EndNote 9 software (Philadelphia, PA, USA).

Authors meticulously handled the removal of study overlaps that could not be processed by EndNote during the screening phase. Subsequently, the two reviewers proceeded with screening and including studies, engaging in comparative analyses and constructive debates to determine the studies for inclusion.

### Data charting process, data items, synthesis of results

The characteristics and type of data to be extracted from the studies were jointly decided by the two reviewers immediately after the study selection phase; the data concerned: the first author, the year of publication, the bibliographic reference, the type of study, the number of teeth or samples being analyzed the identification method investigated, the main results or conclusions of the study. The data were extracted independently by the two reviewers in two different tables and subsequently compared and reported in a third table with three reviewers who verified the correct insertion of the data.

## Results

### Selection of sources of evidence

The research in Science Direct, SCOPUS, PubMed and the Cochrane Library produced a number of records equal to 410. With the removal of duplicates, 334 were obtained; potentially eligible articles totaled 14, of which only 4 fully complied with the criteria of eligibility.

**Fig. 1:**
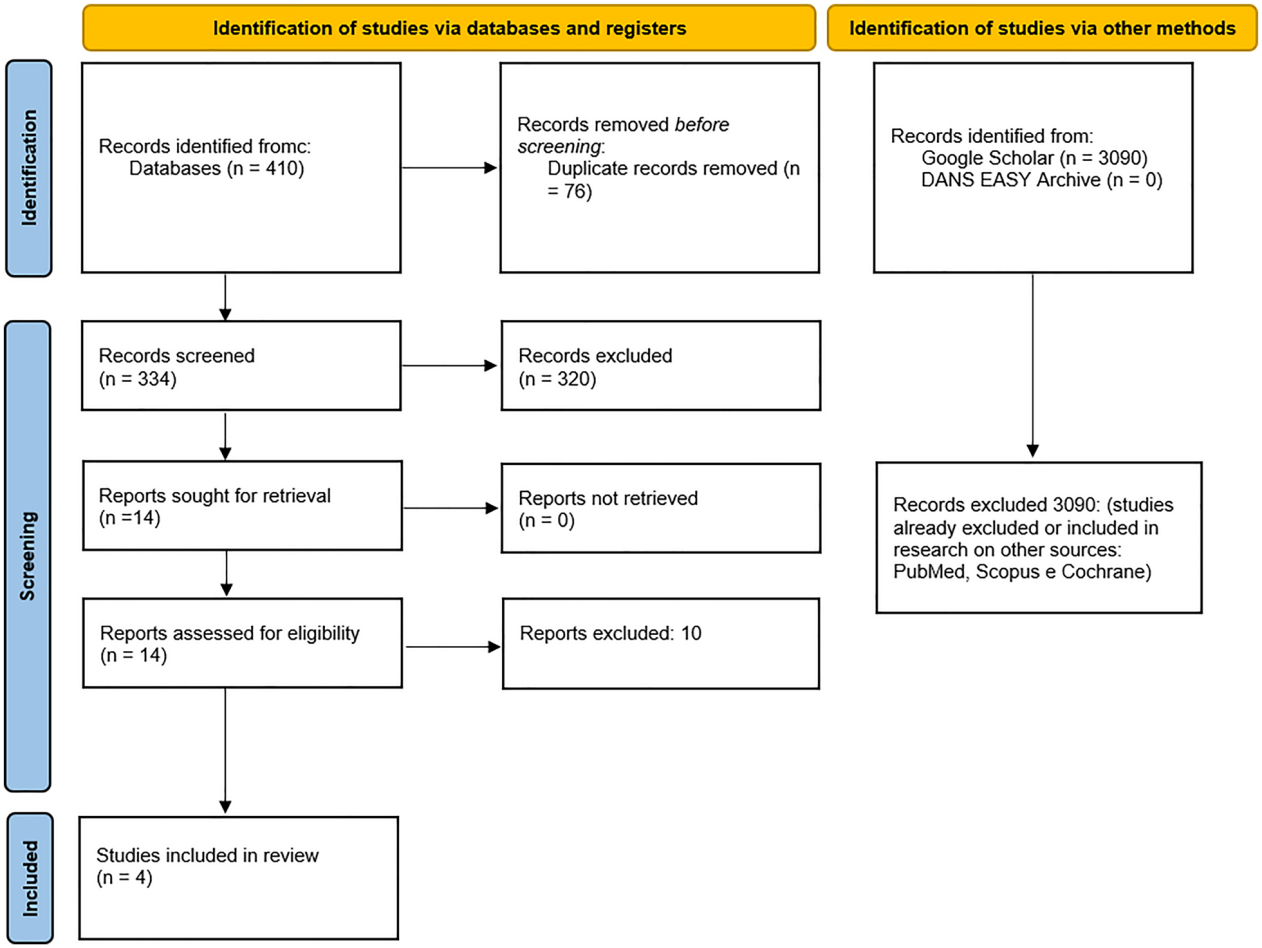
Flowchart of the article selection process (Records identified: PubMed 54, Scopus 127, Science Direct 228, Cocrane Library 1)

In addition, the gray literature analysis (http://www.opengrey.eu, accessed on 19 November 2023, DANS EASY Archive and Google Scholar) and previous systematic reviews did not allow the identification of further additional studies to be included in the quantitative evaluation. The entire procedure for the identification, selection and inclusion of the studies is indicated in the flowchart in Fig. 1.

### Characteristics of sources of evidence, results of individual sources of evidence

A total of four articles were included in the scoping review: Ramenzoni et al., 2006 [7], Arrieta and Line, 2017 [8], Arrieta et al., 2018 [10], Bressan Fogali et al., 2022 [11].

All four studies were conducted by research groups in Brazil, with one study also carried out in Germany [11]. A total of 352 dental elements were examined, with a primary focus on lower incisors, encompassing both central and lateral incisors and totaling 321 specimens. All images underwent processing and manipulation using image software, such as Corel Photo Paint 9, ImageMagick, and ImageJ.

The objective was to highlight internal dental perikymata determined by Hunter–Schreger bands (HSB) for utilization with fingerprint identification software (Verifinger Demo 4.2 SDK/Fingersec). All extracted data are documented in Table 1.

**Table 1:** Data extracted from the four included studies

First autor, data	Country	Tooth	Number	Camera	Software image	Results
Ramenzoni et al., 2006 [7]	Brazil	Central inferior incisors d	30 individuals and 274 central inferior incisors d sample	Pentax MZ-M photo graphic camera and ILFORD 50 ASA (Kodac)	Corel photo paint 9	The HSBs show an image similar to a fingerprint and represent a physiological biometric trait suitable for human identification and verification
Arrieta et al., 2018 [10]	Brazil	Lower incisor teeth	36 lower incisor teeth (24 extracted and 12 photographed in the oral cavity	Canon EOS 5S camera coupled with a InfiniProbe TS-160 objective (Infinity Photo-Optical Company, Boulder, CO)	ImageMagick	Obtaining images of sufficient quality that can be used as biometric identification parameters
Bressan Fogali et al., 2022 [11]	Brazil, Germany	\	31 extracted teeth	\	\	Improvement of image quality
Arrieta and Line, 2017 [8]	Brazil,	Lower central incisors	11 lower central incisors	Canon EOS 5S camera. The ISO was of 200	ImageJ	Improvement of image quality

\ data not available

## Discussion

The following literature review aimed to investigate the role of Hunter–Schreger bands (HSB) in personal identification. Four studies, comprising 352 predominantly lower incisors, were considered for the screening process. All four studies reported data and results on the potential use of dental photographs to visualize HSB, which, after appropriate processing, can be uploaded to fingerprint identification software.

The concept of "biometrics" refers to identification techniques based on physical characteristics [12]. Common biometric identification methods in forensic medicine include DNA analysis [13] and fingerprinting. However, one limitation of these biometric analyses in recognizing cadaveric remains is their susceptibility to near-total obliteration through incineration or burning [14], leaving only small body fragments [15]. In this context, dental enamel, being among the most mineralized tissues, has a greater potential for post-mortem preservation, resisting extreme conditions, such as exposure to high temperatures, pressure, and excessive humidity [16].

HSB analysis is primarily obtained by sectioning dental elements and through electron microscope scans or visualization using polarized light microscopy, causing destruction or irreversible damage to samples, which in some cases are unique.

However, HSB can be visualized on the external surface of the tooth by illuminating it laterally with an external light source, providing an image that, when photographed, exhibits similarities with a fingerprint, displaying ridges and valleys. HSB often bifurcate, and these bifurcations, along with endpoints, represent "minutiae," whose uniqueness can be utilized in algorithms for personal identification [17].

The low contrast between light and dark bands, characteristic of HSB in many teeth, can pose significant challenges in their analysis. Additionally, the area nearest to the light source (medial or distal surface of the tooth) is generally overexposed, while the opposite surface is poorly illuminated. Furthermore, factors such as the reflection of incident light, generating areas of intense brightness in digital images, and the abrupt drop in illumination after light passes through vertical fissures need to be addressed.

These factors can reduce the surface that can be clearly observed, influencing both image quality and analysis reliability.

The bands are predominantly visible on the cervical and middle third of the teeth. The image of Hunter–Schreger bands (HSB) in the tooth proves to be highly tooth-specific, even when compared with the contralateral tooth, as reported by Ramenzoni et al. HSB can be observed through photographic images of teeth heated up to a temperature of 300 ℃ for an hour, after which they cease to be visible due to the incorporation of combustion residues within the enamel. In a study conducted by Ramenzoni, which stored images of 276 lower lateral incisors and photographed the mouths of 30 individuals, it was found that HSB were visible in 95.5% of the lower incisors, and the images were processable through the use of fingerprint identification software (biometrics-based personal identification software—Verifinger Demo 4.2 SDK/Fingersec). The conclusion was that the analysis of dental impressions (photographic HSB9) is non-invasive, accurate, and can be easily performed in automated systems [7].

Arieta et al., in a study conducted on 12 individuals and 36 extracted lower incisors, successfully rendered clear images of HSB by enhancing contrast through the free ImageMagick software, implementing additional commands through Ruby scripts. The images proved suitable for both distal and mesial lighting methods, further enhanced by optimizing algorithms and obtaining optimal parameters from Fogali et al. [11].

The impression of the external tooth surface has been considered using cellulose acetate films, onto which enamel surface ameloglyphics were imprinted, as reported by Manjunath et al. in 2011. In this case, the impression is not precisely determined by Hunter–Schreger bands (HSB) but rather by the different arrangement and orientation of enamel prism groups that protrude onto the external dental surface [18]. The ameloglyphics recorded on acetate films would primarily reflect Retzius' striae [19]. This methodology has been extensively investigated in many studies [20–24]; however, its analysis goes beyond the scope of this review, as ameloglyphics are not necessarily indicative of HSB and are easily mutable, representing an indication of external perikymata of the tooth.

The limitations of this scoping review are mainly represented by the low number of studies on the subject, including only four studies, whose research groups are predominantly Brazilian.

## Conclusions

Nevertheless, within the study's limitations, we can assert that HSB could be used in personal identification, as they are characteristics that are difficult to change and easily detectable. The analysis can be performed through fingerprint software after careful image processing, which currently represents a limitation to the methodology.

## Data Availability

All data generated or analysed during this study are included in this published article.
